# Irruptive mammal host populations shape tularemia epidemiology

**DOI:** 10.1371/journal.ppat.1006622

**Published:** 2017-11-16

**Authors:** Juan J. Luque-Larena, François Mougeot, Beatriz Arroyo, Mª Dolors Vidal, Ruth Rodríguez-Pastor, Raquel Escudero, Pedro Anda, Xavier Lambin

**Affiliations:** 1 Departamento de Ciencias Agroforestales, Escuela Técnica Superior de Ingenierías Agrarias, Universidad de Valladolid, Palencia, Spain; 2 Instituto de Investigación en Recursos Cinegéticos (Consejo Superior de Investigaciones científicas—Universidad de Castilla-La Mancha–Junta de Comunidades de Castilla-La Mancha), Ciudad Real, Spain; 3 Facultad de Medicina, Universidad de Castilla-La Mancha, Ciudad Real, Spain; 4 Centro Nacional de Microbiología, Instituto de Salud Carlos III, Majadahonda, Spain; 5 School of Biological Sciences, University of Aberdeen, Aberdeen, United Kingdom; Nanyang Technological University, SINGAPORE

## Host population dynamics are the key of wildlife zoonotic risk

Infectious diseases affecting humans and involving rodents are rising and ubiquitous. One of every 10 rodent species is a zoonotic host of up to 244 zoonotic pathogens, including bacteria, viruses, helminths, and protozoa [1]. Muroid rodents (rats, mice, voles, gerbils, hamsters) account for 25% of all living mammals, and their high reproductive output and rapid population turnover make them highly permissible amplification agents of zoonotic pathogens [1]. Many muroid populations show strong rates of increase and high-amplitude multiannual fluctuations in abundance (“population outbreaks”), spanning several orders of magnitude. The prevalence of zoonotic pathogens is claimed to be higher in populations that experience outbreaks [1]. Where zoonotic host populations fluctuate in size, considering how such fluctuations contribute to variation in zoonotic disease risk is paramount [2].

Variation in transmission efficiency underpins the dynamics of pathogens [3]. Zoonotic pathogens are often harbored by multiple vector and reservoir species. A precise knowledge of the life cycle and zoonotic transmission routes, and of their variation with host abundance, is therefore essential for understanding the dynamics of zoonotic diseases. Yet surveying the temporal changes in abundance of a few species may suffice to predict zoonotic risk changes. For instance, consideration of changes in the numbers of key hosts and vectors is integral to prevention strategies for zoonotic cholera, dengue, West Nile virus, hantaviruses, or Lyme disease [4]. Rapid population growth in such key species translates into a subsequent increased infection risk to humans. It is thus a research priority to acquire basic epidemiological information about how temporal changes in host abundance modulate zoonotic risk for those wildlife-derived zoonoses that show episodic outbreaks in humans [5].

One infectious disease with highly variable incidence in Europe is tularemia, caused by the etiological agent *Francisella tularensis* subs. *holarctica*, a facultative intracellular gram-negative bacterium of extremely high infectivity and listed as a Class A biothreat agent by the Centers for Disease Control and Prevention (CDC). More than 15,000 human cases were reported from 1997 to 2013 [6], most of which during discrete outbreak episodes separated by interepizootic periods. Up to 250 different animal species are susceptible to infection by *F*. *tularensis* [7], but empirical evidence about transmission routes remains limited. Novel insights from southern Europe may, however, shed light on the dynamics of this highly infectious zoonotic pathogen.

## Aquatic and terrestrial agents of tularemia coexist in nature

**Fig 1:**
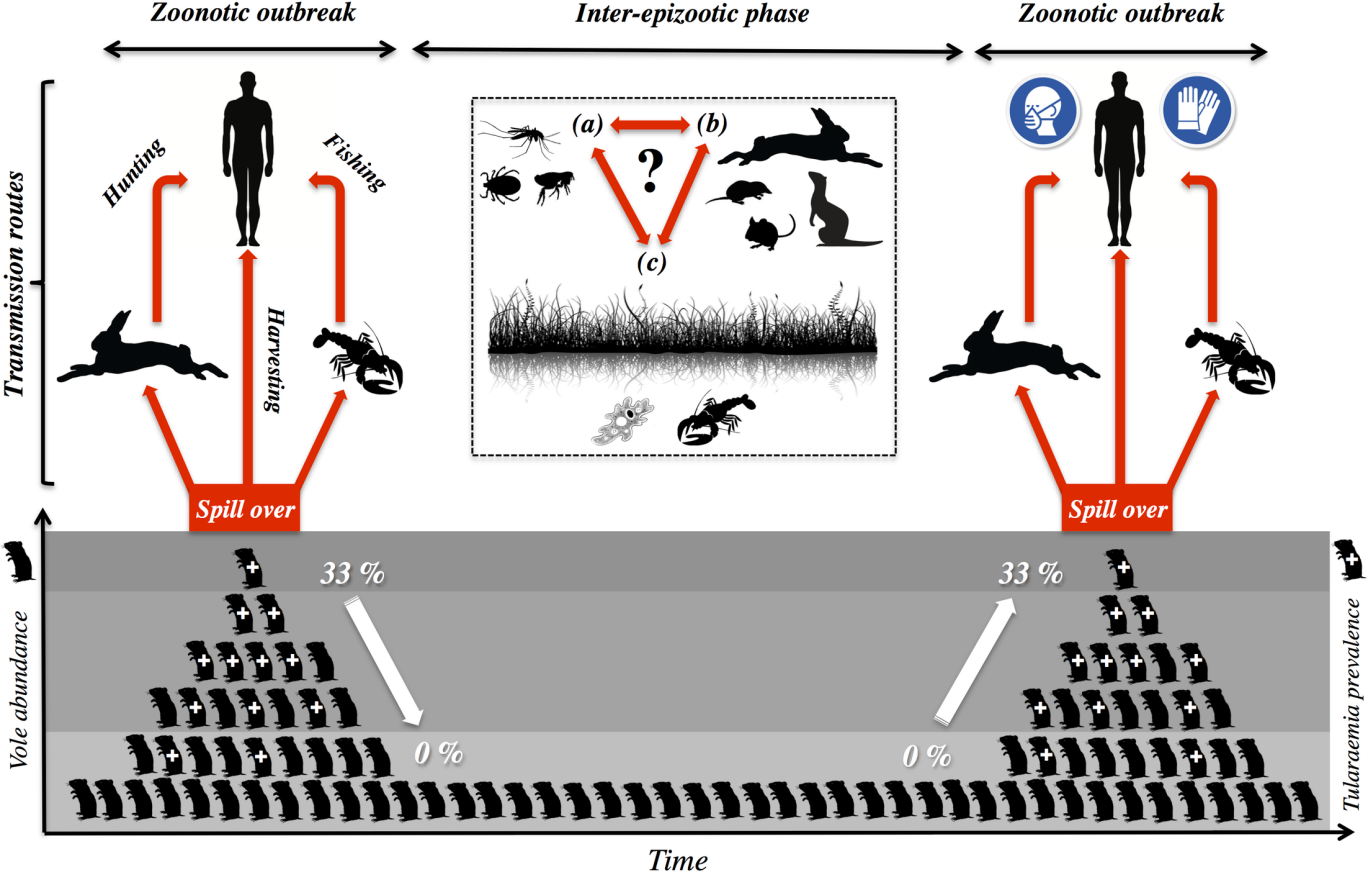
Dynamics of tularemia outbreaks in Northwest Spain. Common voles (*Microtus arvalis*) are key agents for this disease in Northwest Spain (Castilla-y-León region), where outbreaks of tularemia among humans have been endemic in farming landscapes since 1997 (>1,300 cases in 1997–2016). Voles have been identified as a main spillover and amplification agent of tularemia because epizootic and epidemic episodes coincide in time and space with vole outbreaks. When the rodents reach peak densities (>1,000 voles/ha), up to 33% of them are infected with tularemia. Therefore, as vole numbers increase, so does the bacterium in the environment. Transmission routes of tularemia to humans during zoonotic outbreaks include (i) direct contact with wildlife species such as hares or crayfish, which coexist with voles in the same habitats, and (ii) trough inhalation during the harvesting of vole-infested crop fields. At low vole densities, the bacterium is not found among the rodents, indicating that, between vole outbreaks, populations of *F*. *tularensis* subs. *holarctica* may remain at lower numbers, associated with some yet-unknown reservoirs. Enzootic cycles in local wildlife other than voles, including hematophagous arthropods (a) and other small- and medium-sized mammals, (b) may also contribute to sustaining the bacteria in the environment during inter-epizootic periods. Water is a main habitat for reservoir candidates (c) because it is a well-known favorable habitat for tularemia (most especially in these semiarid landscapes of Northwest Spain).

It has recently been suggested that tularemia has both a terrestrial and a distinct aquatic life cycle in Europe, owing to terrestrial and aquatic organisms having been implicated as vectors of transmission to humans [6]. The former involves primarily lagomorphs (rabbits, hares), terrestrial rodents, and ticks, whereas the aquatic cycle involves mosquitoes and their larvae, as well as semiaquatic rodents [6]. Recent evidence from Spain [8, 9]—where both hypothetical life cycles are said to occur [6]—is, however, compatible with a single, more unified life cycle, including coexisting zoonotic hosts and either terrestrial or aquatic amplification (Fig 1).

Human tularemia is endemic in Spain, with 1,386 clinical cases described between 1997 and 2016 by the National Network of Epidemiologic Surveillance of Spain (Red Nacional de Vigilancia Epidemiológica [RENAVE], Instituto de Salud Carlos III, Madrid, Spain). Virtually all cases (>1,300) have been described in the region of Castilla-y-León, Northwest Spain. Additionally, an isolated outbreak (19 cases) of human ulceroglandular tularemia was reported in central Spain in 1998 [10]. The latter was associated with manipulation of non-native crayfish (*Procambarus clarkii*) in a water reservoir, consistent with a role for aquatic zoonotic vectors. Most instances of human-acquired tularemia in Castilla-y-León occurred during 2 larger outbreaks recorded in 1997–1998 (585 human cases) and 2007–2008 (639 human cases; RENAVE) and were associated with (i) contact with Iberian hares (*Lepus granatensis*) or common voles (*M*. *arvalis*; ulceroglandular and glandular forms; 71% of cases in 1997–1998) and (ii) inhalation during harvesting of crops invaded by common voles (pneumonic and typhoidal forms; 65% of cases in 2007–2008) [11]. In 2014, 95 human cases of tularemia were also confirmed in Castilla-y-León, coinciding with a regional increase of vole numbers [8]. Terrestrial vectors such as voles and hares evidently transmit this zoonosis, but a human clinical case involving aquatic crayfish handling was also described in the same region in 2001 during an interepizootic period [12], implying that the bacterium is also present in water. Therefore, both aquatic and terrestrial agents of tularemia coexist in nature in Northwest Spain.

## Irrigation has provided aquatic reservoirs and a grass-loving amplification agent for tularemia in Spain

The climate of Castilla-y-León features hot and dry summers and a hostile environment for the survival of *F*. *tularensis* on land [13]. Mesic habitats also restrict the abundance of terrestrial reservoirs such as ticks. *F*. *tularensis* can, however, survive in water [13], including through the parasitism of protozoans that act as reservoir hosts [14]. Indeed, proximity to water is associated with higher incidence rates of tularemia in northern Europe [15]. Crucially, the bacterium is not amplified in water, and its life cycle requires mammalian hosts for amplification [13, 16]. The surface area of irrigated crops doubled in the agroecosystem of Castilla-y-León between the 1970s and 1990s [17], prior to the local emergence of tularemia [8, 11]. Not only does the extensive network of irrigation canals and ditches provide suitable conditions for an aquatic persistence of tularemia but the presence of irrigated crops has also triggered the colonization of millions of hectares of hitherto unoccupied habitats by common voles [17]. Common voles have been identified as a main spillover and amplification agent of tularemia because (i) epizootic and epidemic episodes coincide in time with vole outbreaks [8], and (ii) at peak density (>1,000 voles/ha), up to 33% of live voles are infected with tularemia [9]. What was an inhospitable semiarid landscape has become a suitable environment for the spread and maintenance of tularemia as an endemic disease in Castilla-y-León.

## Fluctuating mammalian populations shape tularemia epidemiology

It has long been accepted that fluctuations in the abundance of wild herbivorous mammals (hares, voles) play a key role in tularemia epidemiology in European countries accumulating the largest numbers of clinical cases (i.e., Sweden, Finland, Czech Republic, Hungary, Spain) [6, 8, 14, 16, 18]. Irrespective of whether human infection is vectored by ticks or mosquitoes, contact with harvested fish and game, or contaminated water, air, or food, epidemics coincide temporally with increases in the abundance of an *F*. *tularensis*–mammalian host. In Sweden, peaks in vole and hare populations and outbreaks of tularemia in humans were simultaneously recorded during the 1960s and 1970s [18]. In the Novosibirsk region (Russia), the number of human cases of tularemia was also correlated with the density of the water vole population between 1956 and 2000 [19]. The high-amplitude multiannual fluctuations in the abundance of muroid rodents and hares are wholly consistent with irruptive increases of tularemia prevalence among these vector hosts, leading to rapid amplification of the bacterium and contamination of the environment as hares and voles succumb to tularemia.

There is also evidence that the contribution of lagomorphs and rodents may change over time, according to their abundance. In Saskatchewan (Canada), contact with lagomorphs was the common route for human infection before the 1950s, while rodents became of greater importance afterwards [20]. Extensive serological surveys among human populations in Castilla-y-León showed practically no evidence of *F*. *tularensis* (prevalence of antibodies <0.19%) until 1997 [21], coinciding with the final stage of the colonisation of the agroecosystem by common voles [17], but the early human outbreak (1997–1998) was associated with handling of shot hares and an episode of massive hare mortality, which led to enduring low hare abundance [11]. Subsequent human tularemia outbreaks (2007–2008, 2014) have been associated with periods of superabundance of common voles [8], which attain much higher abundance and biomass than hares and rabbits. The empirical evidence suggests that pulses of abundance of hosts that amplify the bacterium within host populations and ultimately contaminate the terrestrial and aquatic environments may be of greater epidemiological significance than host taxonomy, probably associated with the low competent host specificity of tularemia.

## Tularemia surveillance must target unstable mammalian host populations

The “One Health” concept advocates a broad view of medicine for the successful development of policies and practices that reduce the impact of zoonoses through targeted surveillance and strategic prevention [5]. Monitoring of populations of key epidemiological agents such as voles and hares should be central to prevention strategies. Vole surveillance programs are already implemented in the Castilla-y-León region, showing a degree of predictability to vole populations fluctuating with region-wide outbreaks every 5 years [22]. Extending vole monitoring to include tularemia, particularly during increasing and outbreak population phases, would provide crucial data to parameterize spatial–temporal models of disease risk and help predict when people engaging in nonoptional (e.g., crop harvesting) and optional (e.g., hare hunting, crayfish fishing) risky activities should adopt appropriate risk-minimizing techniques (e.g., farmers using breathing masks during summer harvests in vole outbreaks, hunters and fisherman using gloves during hare butchering or crayfish cleaning; Fig 1).
